# Self-reported urinary incontinence and factors associated with symptom severity in community dwelling adult women: implications for women’s health promotion

**DOI:** 10.1186/1472-6874-13-16

**Published:** 2013-04-08

**Authors:** Vidya Seshan, Joshua Kanaabi Muliira

**Affiliations:** 1 College of Nursing, Sultan Qaboos University, P. O. Box 66 Al Khod, Muscat 123, Oman

**Keywords:** Risk factors, Women, Attitude, Urinary incontinence, Predictors, Prevalence

## Abstract

**Background:**

Urinary incontinence (UI) continues to affect millions of women worldwide and those living in resource poor settings seem to be more affected. The purpose of this study was to determine the prevalence of UI and factors associated with UI symptom severity (UISS) among women in a selected district in India.

**Methods:**

A cross-sectional design was used to collect data from a sample of 598 community dwelling women in the age range of 20 to 60 years. Data was collected using a questionnaire survey of participants who were found in their homes.

**Results:**

The prevalence of UI was 33.8% and the majority of women had negative attitudes about the condition. For instance most women were in agreement with statements such as: UI cannot be prevented or cured (98%); women with UI are cursed (97%); women are not supposed to tell anyone about the problem (90%) and others. Of the 202 women with self-reported UI, the majority reported having moderate UISS (78%) and others rated the symptoms as mild (22%). The woman’s age at first birth (p<.01) was negatively associated with UISS, while the number of pregnancies (p<.01) and weight of the largest baby ever delivered (p<.01), were positively associated with UISS. The weight of the largest baby delivered had the strongest impact on predicting UISS.

**Conclusions:**

Many community dwelling women are suffering from UI at proportions which warrant significant public health consideration. Therefore public health programs to prevent UI or worsening of symptoms are required and should emphasize health education, because of the pervasive negative attitudes among affected and unaffected women. The predictors reported here can be used to priotize care for affected women and to encourage early uptake of health actions and behaviors that promote pelvic floor strengthening in at risk women who may be reluctant to disclose UI.

## Background

Urinary incontinence (UI) is a common condition that affects millions of people during adult life [1]. Recent statistics show that 348 million people worldwide experienced any type of UI in 2008 and this number is projected to increase to 423 million by the year 2018 [2]. Generally UI is more common in women and the burden of this condition is greatest in developing counties of Asia, South America and Africa [2]. In 2008 a total of 250 million women in Asia, South America and Africa were affected by UI and this number is expected to increase to approximately 303 million by 2018 [2]. The impact of UI is much higher among the middle aged and older women, and in these categories of women, its prevalence is estimated to be 40% and 50%, respectively [3].

Women are at a high risk of UI mainly because of the damage to the pelvic floor caused by pregnancy and the child birth process [4-6]. Pregnancy alone leads to mechanical and hormonal changes which impair pelvic floor muscle strength. The effects of pregnancy can be extended by vaginal delivery and this increases the risk of getting UI. The childbirth process has the potential of damaging the muscle and nerves of the pelvic floor, which control of urine flow [7-9]. The women who deliver large babies or by forceps (instrumentation) through the vaginal route, are at a even higher risk of UI. Delivering a large baby and through instrument assisted methods can cause major trauma to pelvic floor muscles and nerves, and this significantly reduces perineal floor strength [10,11]. Therefore the key aspects that enhance the risk of UI in women are excessive stretching, multiple deliveries and other situations that repetitively cause stretching and trauma to the pelvic floor tissues [3,12-15].

Recent studies have identified genes that encode for components of the extra cellular matrix and these are thought to predispose some individuals to UI [3,16]. Analysis of endopelvic fascia biopsies in women with UI, shows that their tissues have increased degradation of nascent collagen and collagenolytic activity [16]. These findings indicate a strong link between genetics and risk for UI. But in many resource poor settings the common factors that have been reported to be associated with UI in women include standards of living, poor nutrition (causing poor tissue tensile strength), anemia and regular physical heavy work [12]. The other factors include age, obesity, and menopause [12]. It has to be noted that for a woman to start experiencing actual UI, multiple factors related to her obstetrical, gynecological, genetic and social well-being come together to synergistically contribute to the outcome [15].

Therefore many women in developing and developed countries are still facing the risk of or problem of UI and there are several factors associated with it, although these may be different in different settings [12,17]. There is need to continuously study the UI prevalence and associated factors as both of these are likely to change as countries economy and standards of living change. Such studies enable health care providers to design effective interventions that can help to curtail the negative impact of UI on the quality of life of affected women and families [18]. The purpose of this study was to determine the prevalence of UI in community dwelling women and the factors associated with UI symptom severity (UISS). The definition of UI recommended by the International Continence Society (complaint of any involuntary leakage of urine) was used in the study [19].

## Methods

### Data collection procedures

The study was conducted in Coimbatore South, a division of Coimbatore district in Tamilnadu state of India. The district has six administrative divisions (Coimbatore South, Coimbatore North, Mettupalayam, Sulur, Pollachi and Valparai) and an estimated population of 4,271,856 people (49% female). Coimbatore South has a population of 1,332,789 (681,758 males and 651,031 females) and is the most densely populated division of Coimbatore district. After obtaining approval to conduct the study from the research committee of Vinayaka Missions University, the researchers collected data by approaching homes on a door to door basis in Coimbatore South. The researchers collected the data on weekdays after office hours and during weekends, and this helped to ensure that both employed and unemployed women had a chance of being selected to be in the study.

Women found at home were requested to be participants in the study if they met the inclusion criteria of: age between 20 to 60 years; not antenatal or postnatal mothers (who delivered within the past 6 months). In homes where the woman met the inclusion criteria and agreed to participate in the study, the study purpose and procedures were explained before a written consent form was administered to them to get their consent. After completing the consent form, participants were asked to complete a questionnaire which required information about their demographic characteristics, obstetric characteristics and urinary incontinence (UI). The women were asked to answer to the question “Do you have urinary incontinence?” The participants who answered “yes” on this question (considered as having UI) were required to complete the UISS and attitude towards UI section. The participants who answered “no” were required to complete only the attitude towards UI section. The study sample was comprised of 598 women.

### Study instrument

The questionnaire used for data collection was developed by the researchers after extensive review of the literature on UI and associated factors in women. The questionnaire was comprised of a section on demographic characteristics, obstetric characteristics, attitude towards UI and UISS. The contents of the questionnaire were reviewed by experts in the field of Nursing, Obstetrics and Gynecology, Urology, General Medicine, Sociology and Biostatistics. Twelve experts reviewed the tool for content validity and gave feedback about each item regarding its precision, feasibility and appropriateness on a scale of 1 to 4 (1= very inappropriate, 2= inappropriate, 3= appropriate, and 4= very appropriate). Items were considered as valid if they received a score of 3 or 4 points from at least 8 of the 12 experts. The questionnaire was modified according to the suggestions of experts before double translation into Tamil and English languages to establish semantic equivalence. The demographic characteristics included aspects such as age, marital status, education, occupation, and parameters to calculate body mass index (BMI).

The obstetric characteristics included age at marriage, maternal age at first birth, number of pregnancies, number of abortions, number of live births, type of delivery on the last baby, menopause status and weight of the largest baby ever delivered. The items about attitude towards UI, required participants to respond to ten statements with answers such strongly disagree, disagree, neutral, agree and strongly agree. To determine whether participants had UI or not, they were asked to respond to the question “Do you experience any involuntary leakage of urine in your daily life”. The participants who responded “yes” were categorized as having UI and those who responded “No” as not having UI. The section on UISS consisted of 22 items asking participants who reported having UI to rate the severity of their UI symptoms. For instance participants responded to items such; “I experience leaking of urine after urinating”; “I have Bad odor due to UI”; “How often does this type of urinary leakage occur?”; “Approximately how much urine do you lose each time?” and others. The participants’ responses on each item were rated on a Likert scale as: “never” = 0, “occasionally” = 1, “often” = 2 and “always” = 3. The items about frequency of UI, amount of urine leakage, activities leading to UI and length of time with UI were not scored using the Likert scale, but with a scale indicating level of severity according to the reported number, amount or intensity of the activity. The total possible score on the severity of UI symptoms section ranged from 0 to 75.The total score of each participant were categorized as mild UI (score=0-25), moderate UI (score =26-50) and severe UI (score =51-75).

There was no clinical examination or uro-dynamic testing conducted for the participants. The reliability of the UI symptom severity section and attitude section were assessed using Cronbach’s alpha and was found to be 0.73 and 0.82, respectively. Data analyses were conducted using SPSS for Windows (SPSS-17.0). Prevalence’s of UI was determined using measures of distribution. Chi-square statistic was used to examine differences in demographic and obstetrical characteristics of participants affected and those not affected by UI. Correlation and multiple linear regression analyses were conducted to examine the factors associated with severity of UI symptoms and various predictors. In all statistical analyses p ≤ .05 were considered significant.

## Results

### Socio-demographic characteristics of the participants

The majority of participants recruited for the study were above the age of 30 years. The results presented in Table 1 also show that the participants were mostly of low education level (secondary school and below = 67%), working in low paying jobs (house helper or office clerk = 24%) or as house wives (55%). A large number of participants were married (85%) and found to have a normal body mass index (70%).

**Table 1:** Demographic characteristics of the participants

**Variables**	**Category**	**N=598 frequency (%)**
**Age in years**	20-29	177 (29.6)
	30-39	186 (31.1)
	40-49	114 (19.1)
	50-60	121 (20.2)
**Body Mass Index**	Underweight (<18.5)	34(5.7)
	Normal (18.8-24.9)	420 (70.2)
	Over weight (25–29.9)	114 (19.1)
	Obese (>30)	30 (5.0)
**Marital Status**	Single	21 (3.5)
	Married	509 (85.1)
	Widow	68 (11.4)
**Educational Level**	No formal Education	27 (4.6)
	Primary School level	233 (38.9)
	Secondary School level	139 (23.2)
	Graduate Level	175 (29.3)
	Postgraduate Level	24 (4.0)
**Occupation**	House Wife	332 (55.5)
	House Helper or Maid	74 (12.4)
	Office Clerk	68 (11.4)
	School teacher	34 (5.7)
	Self Employment	47 (7.9)
	Professional	43 (7.1)

### Prevalence and severity of urinary incontinence

Of the 598 participants recruited in the study, a total of 202 (33.8%) reported that they have UI and 396 (66%) denied having UI (see Table 2). Among those who reported having UI (n=202), the majority rated the severity of their symptoms as moderate (78%). The prevalence of UI among women included in this study was at proportions equivalent to a major public health problem.

**Table 2:** Self-reported urinary incontinence and severity of urinary incontinence symptoms

**Variables**	**Response**	**N=598 frequency (%)**
Do you experience any involuntary leakage of urine in your daily life?	Yes	202 (33.8)
	No	396 (66.2)
Severity of Urinary Incontinence Symptoms	No symptoms	396 (66.2)
	Mild (21.7% of 202)	44 (7.4)
	Moderate (78.2% of 202)	158 (26.4)
	Severe (0% of 202)	0 (0)

### Characteristics of women affected by urinary incontinence

As shown in Table 3, the participants with UI (n= 202) were mostly those in the age group of 50 to 60 years (43%); with low levels of education (primary school or lower level of education =56%); working at home as housewives (57%) or house maids/helpers (16%); and with a body mass index (BMI) above the normal limit (52%). It is important to note that despite having UI, majority of the affected women were still married (78%). The results in Table 4 show that the participants with UI mostly got married (65%) and gave birth to their first child (52%) at an age of less than 20 years (52%); delivered their largest baby by vaginal delivery with an episiotomy (63%); had been pregnant more than 3 times (65%); and had one or more abortions in the past (79%). Bivariate analysis (see Table 3 and 4) show that there were significant differences in demographic and obstetrical characteristics of women with and without UI.

**Table 3:** Comparison of demographic characteristics of participants with and without unitary incontinence

**Characteristic**	**Category of participant (N =598)**		**Pearson *X***^***2***^	**p value**
	**Women with UI (n=202) frequency (%)**	**Women with-out UI (n=396) frequency (%)**		
**Age in years**	(M =46.6, SD =9.46)	(M =32.9, SD =10.02)		
20-29	6 (3)	171(43.2)	179.67	< .01
30-39	46 (22.8)	140(35.4)		
40-49	64 (31.7)	50(12.6)		
50-60	86 (42.6)	35(8.8)		
**Body Mass Index (BMI)**	(M=25.53, SD =3.79)	(M =22.01, SD =2.76)		
Underweight (< 18.5)	4 (2)	30(7.6)	131.21	< .01
Normal (18.8 -24. 9)	94 (46.5)	326(82.3)		
Over weight (25–29.9)	78 (38.6)	36(9.1)		
Obese ( > 30)	26 (12.9)	4(1)		
**Marital Status**				
Single	0 (0)	21(5.3)	40.30	< .01
Married	158 (78.2)	351(88.6)		
Widow	44 (21.8)	24(6.1)		
**Level of Educational**				
No formal education	15 (7.4)	12(3.1)	31.06	< .01
Primary School level	99 (49)	134(33.8)		
Secondary School level	26 (12.9)	113(28.5)		
Graduate level	58 (28.7)	117(29.5)		
Postgraduate level	4 (2)	20(5.1)		
**Occupation**				
House wife	116 (57.4)	216(54.5)	13.21	< .01
House Helper or Maid	32 (15.8)	42(10.6)		
Office Clerk	22 (10.9)	46(11.6)		
School Teacher	8 (4)	26(6.6)		
Self Employed	18 (8.9)	29(7.3)		
Professional	6 (3)	37(9.3)		

*M*: Mean; *SD*: Standard deviation; *UI*: Urinary incontinence; *X*^*2*^: Chi-square.

**Table 4:** Comparison of obstetrical characteristics of participants with and without unitary incontinence

**Obstetrical characteristic**	**Women with UI (n=202) frequency (%)**	**Women with-out UI (n=396) frequency (%)**	**Pearson *X***^***2***^	**p value**
**Age (in years) at marriage**	(M =19.23, SD =3.49)	(M =21.75, SD =3.39)		
< 20	132 (65.3%)	99 (25%)	97.50	< .01
20-25	64 (31.7%)	239 (60.4%)		
25-30	6 (3%)	33 (8.3%)		
>30	0 (0%)	4 (1%)		
**Age (in years) at first birth**	(M =21.06 , SD =3.30)	(M =23.71, SD =2.88)		
< 20	106 (52.5%)	39 (9.8%)	143.14	< .01
20-25	72 (35.6%)	236 (59.6%)		
25-30	22 (10.9%)	73 (18.4%)		
>30	2 (1%)	4 (1%)		
**Number of Pregnancies**	(M =3.26, SD =1.28)	(M=2.23, SD =2.27)		
1 - 2	70 (34.7%)	266 (67.1%)	168.46	< .01
3 - 4	92 (45.6%)	79 (20%)		
5 - 6	40 (19.9%)	4 (1%)		
**Number of Abortion**	(M =0.92, SD =0.794)	(M =0.91, SD =2.51)		
No abortion	62 (30.7%)	294(74.3%)	156.25	< .01
1 - 2	134 (76.3%)	72(18.2%)		
3 - 4	6 (3%)	3(0.8%)		
**Number of Deliveries**	(M = 2.36, SD =0.88 )	(M= 2.28 , SD =2.26)		
1- 2	126 (62.4%)	304(76.5%)	91.41	< .01
3 - 4	72 (35.6%)	40(10.1%)		
≥ 5	2 (1%)	4(1%)		
**Type of delivery for last Baby**				
Vaginal with episiotomy	134 (63.3%)	232(58.6%)	67.13	< .01
Assisted vaginal delivery	60 (29.7%)	54(13.6%)		
Caesarean Section	6 (3%)	58(14.6%)		
**Weight of Largest Baby (Kg)**	(M = 2.33, SD =0.43)	(M = 2.5, SD =0.49)		
2-3	110 (54%)	262(66.2%)	49.89	< .01
3-4	10 (6%)	84(21.2%)		
>4	80 (40%)	0(0%)		
**Woman is in Menopause**				
Yes	88 (43.5%)	34(8.6%)	90.82	< .01
No	114 (56.4%)	362(91.4%)		

*M*: Mean; *SD*: Standard deviation; *UI*: Urinary incontinence; *X*^*2*^: Chi-square.

### Participants attitude towards urinary incontinence

The attitudes of women with and without UI, towards UI were mostly negative. As shown in Table 5, a large number of women with and without UI (more than 80%) reported that they agree with statements such as: accidental loss of urine is a common problem that everyone woman faces; women should not go for social events if they have UI; there is no treatment available for UI; UI cannot be prevented or cured; and others. On a separate item [not included in the table] women affected by UI reported that the measures they take to reduce the UI symptoms were: going to toilet to urinate every 2 hour (63%); reducing fluid in-take including water (53%); and not revealing their problem to any one (65%).

**Table 5:** **Participants attitudes towards urinary incontinence** (UI)

**Statement**	**With UI (n=202) frequency (%)**		**Without UI (n=396) frequency (%)**	
	**Agree**	**Disagree**	**Agree**	**Disagree**
Accidental loss of urine is a common problem that everyone woman faces	202 (100)	0 (0)	361 (91.2)	35 (8.8)
Women should not go for social events if they have UI	202 (100)	0 (0)	336 (84.8)	60 (15.2)
Women are not supposed to tell anyone if they have UI	199 (98.5)	3 (1.5)	342 (86.4)	54 (13.6)
A woman who has UI should avoid sexual intercourse	196 (97)	6 (3)	379 (95.7)	17 (4.3)
Life style changes has no effect on symptoms and risk of getting UI	200 (99)	2 (1)	386 (97.5)	10 (2.5)
Restricting fluid intake is a good thing to do when one has UI	196 (97)	6 (3)	360 (90.9)	36 (9.1)
Exercise is one of the main causes of UI	194 (96)	8 (4)	370 (93.4)	26 (6.6)
Urinary Incontinence cannot be prevented	196 (97)	6 (3)	391 (98.7)	5 (1.3 )
Women who have Urinary Incontinence are cursed	196 (97)	6 (3)	386 (97.5)	10 (2.5)
Urinary Incontinence cannot be cured	198 (98)	4 (2)	387 (97.7)	9 (2.3)

### Factors associated with urinary incontinence symptom severity

Multiple linear regression analysis was used to develop a model for predicting UI symptom severity (UISS) among affected women using objective parameters such as age at first birth, number of pregnancies and weight of the largest baby ever delivered. Basic descriptive statistics and regression coefficients are shown in Table 6. Each of the predictors had a significant (p < .01) correlation with UISS, but only age at first birth had a negative relationship. The correlation statistics show that UI symptoms tend to be more severe in women with a high number of pregnancies, delivered their first birth at a very young age and who have given birth to large babies. The age at first birth, number of pregnancies and weight of largest baby ever delivered predictors had significant (p < .01) partial effects in the full model. The three predictors model was able to account for 44% of the variance in women’s self reported UISS, F = (3, 198) = 21.93, p  ≤ .000, R [2] = .442. Based on this analysis we have recommended an early health education and health promotion interventions in at risk women to prevent UI or worsening of symptoms in those that may be already affected.

**Table 6:** Factors associated with self-reported urinary incontinent symptoms (N=202)

	**Pearson r (2-tailed)**				**b**	**β**
**Variable**	**WLB**	**NOP**	**AFB**	**Urinary incontinence symptom severity**		
Age at First Birth (AFB)				-.353**	-.037**	-.217
Number of Pregnancies (NOP)			-.233**	.386**	.112**	.268
Weight of Largest Baby (WLB)		.244**	-.256**	.576**	.506**	.450
					Intercept = 0.436	
Mean	2.33	3.26	19.23	29.9	R^2^= .44	
Standard Deviation	0.43	1.28	3.49	5.1	ΔR^2^ =.02	

**: p< 0.01; R: multiple correlation coefficient; b: Unstandardized Coefficients; β: Standardized Coefficients.

## Discussion

The findings of this study show that UI affects at least 3 out of 10 community dwelling women in Coimbatore South division of Coimbatore district in Tamilnadu state of India, and this prevalence (33.8%) is higher than the rates reported generally for developing countries (28.7%) [12,17]. The prevalence of UI in the current study is also higher than what has been reported among comparable groups women (in terms of social economic status) in developed countries such as native Indians in USA (15.4%) [20]. Therefore in developing countries such as India, chronic and less life threatening UI, is still impacting women’s health at proportion that require public health interventions. The current study also emphasizes that interventions to prevent UI or alleviate the impact of UI on affected women’s health is challenging because of the negative attitudes.

The majority of women sampled in the current study (with and without UI) had attitudes towards UI which were mostly negative and these could be seen in their agreement with statements such as: accidental loss of urine is a common problem that everyone woman faces; women should not go for social events if they have UI; there is no treatment available for UI; and UI cannot be prevented or cured. These negative attitudes are likely to impede women affected by UI from seeking health care or from engaging in preventive and health promoting activities. The negative attitudes of women may also prevent them from reporting UI and associated symptoms to the health providers. It is therefore possible that the prevalence reported by women in this study is an underestimation of a big problem. We recommend that interventions to reduce the prevalence of UI in resource poor settings have to start with deliberate targeted health education and health promotion interventions in at risk women focusing on prevention of UI and how to alleviate symptoms in those that may be already affected.

Apart from underestimation of the problem, attitudes unfavorable to reporting or seeking care for UI also make it difficult for health care providers to advocate for resources and programs for UI prevention and early diagnosis, which are critical steps in health promotion. In challenging situations like these health care providers have to use evidence based information to target at risk women during assessment, health education and other interventions. But in order to target at risk women health care providers have to be equipped with knowledge of objective predictors. The predictors of UISS indentified by the current study (woman’s age at first birth, number of pregnancies, and weight of the largest baby ever delivered) are part of the health history commonly elicited from female patients, but are not given a lot of emphasis during care planning. The three factors can be easily determined during health assessment and can inform the health care providers of women whom they need to ask about UI symptoms. Getting the women to talk and report UI is a very important starting point in settings where women have unfavorable attitudes. Because once women start talking and reporting UI early diagnosis and low cost interventions can be implemented to enhance quality of life and symptom relief.

The other commonly reported predictors of UI such as age, menopause, and BMI [21] were not found to be significant in this study. In other studies BMI has been reported as a major factor in worsening of UI because it imposes persistent weight on the pelvic tissues causing chronic strain, stretching and weakening of the muscles, nerves, and other structures [22,23]. In our study, the weight of the largest baby delivered had the strongest impact on predicting UISS. Using the objective predictive factors identified in this study, healthcare providers can prioritize care and interventions to help affected women, especially when faced with a large number of patients with UI. Women found to have UI symptoms can be advised and health educated about available interventions that reduce severity of symptoms. Available evidence show that low cost self-directed interventions such as pelvic floor muscle exercises can bring about symptom relief or periods of resolution over time [18].

Other health promoting approaches such as self management by risk factor modification also have shown potential benefits such as reduction in symptom severity, improved self efficacy and incontinence related quality of life. Using the factors identified in this study, health care providers can provide targeted health education on self care practices such as performing Kegel’s exercises and other techniques that promote perineal health and increase self care efficacy as part of their care plan to reduce in UI symptoms.

### Study limitations

The results of the study have to be interpreted with caution because the study had limitation in the following aspect: reliability of data on UISS collected using self-report method and unstandadized questionnaire; determining UI status by self report and based on only one item of “do you experience any involuntary leakage of urine in your daily life”; and convenient sampling.

## Conclusions

Generally prevention and treatment of UI for the increasing number of those affected with this chronic condition, has received little public health attention. Although the current study has some limitations, it enhances our understanding of the predictors of UISS and these can be used to target at risk women for early diagnosis, preventive and health promoting interventions for affected women. Some of the predictors are modifiable and can be addressed as part of other health promotion programs related to reproductive health and family planning. The study also highlights that as the developing countries become more affluent the cultures and attitudes of women towards certain health problem such as UI are lagging behind. Therefore individuals affected by such conditions should be educated by those healthcare providers regularly associated with their care, on the risk factors and health promoting actions that can be taken to reduce their risk for prevalent chronic conditions such as UI.

## Competing interest

The authors declare that they have no conflict of interest with the material presented in this paper.

## Authors’ contributions

VS developed the research proposal, designed the instrument, and collected data in the field sites, analyzed and wrote the draft manuscript. JKM contributed to the study design, assisted with survey instruments, analyzed data, drafted manuscript and contributed to final version of manuscript. Both authors read and approved the final manuscript.

## Pre-publication history

The pre-publication history for this paper can be accessed here:

http://www.biomedcentral.com/1472-6874/13/16/prepub
